# Tailoring Multi-Level Structural and Practical Features of Gelatin Films by Varying Konjac Glucomannan Content and Drying Temperature

**DOI:** 10.3390/polym12020385

**Published:** 2020-02-08

**Authors:** Dongling Qiao, Zhong Wang, Chi Cai, Song Yin, Hong Qian, Binjia Zhang, Fatang Jiang, Xiang Fei

**Affiliations:** 1 Glyn O. Phillips Hydrocolloid Research Centre at HBUT, School of Food and Biological Engineering, Hubei University of Technology, Wuhan 430068, China; 2 Sichuan Sanlian New Materials CO., LTD., Chengdu 610041, China; 3 Group for Cereals and Oils Processing, College of Food Science and Technology, Key Laboratory of Environment Correlative Dietology (Ministry of Education), Huazhong Agricultural University, Wuhan 430070, China; 4 Faculty of Engineering, University of Nottingham, Nottingham NG7 2RD, UK

**Keywords:** konjac glucomannan/gelatin film, multi-level structure, practical features

## Abstract

Here, we tailored the multi-level structural and practical (mechanical/hydrophilic) features of gelatin films by varying the konjac glucomannan (KGM) content and the film-forming temperatures (25 and 40 °C). The addition of KGM apparently improved the mechanical properties and properly increased the hydrophilicity. With the lower temperature (25 °C), the increase in KGM reduced the gelatin crystallites of films, with detectable KGM–gelatin interactions, nanostructures, and micron-scale cracks. These structural features, with increased KGM and negligibly-occurred derivatizations, caused initially an insignificant decrease and then an increase in the strength, with a generally-increased elongation. The higher temperature (40 °C) could reduce the strength and slightly increase the elongation, related to the reduced crystallites of especially gelatin. With this higher temperature, the increase in KGM concurrently increased the strength and the elongation, mainly associated with the increased KGM and crystallites. Additionally, the increase in KGM made the film more hydrophilic; the multi-scale structural changes of films did not dominantly affect the changing trend of hydrophilicity.

## 1. Introduction

Biopolymer-based materials have attracted enormous attention in a wide range of fields, such as food packaging, which is crucial in guaranteeing food hygiene and prolonging the shelf life for perishable food items [1]. Moreover, biopolymers can be eco-friendly, renewable, and abundant, and thus are desired resources for the design and production of biodegradable materials, being alternatives for petroleum-based materials [2,3]. Among the most popular biopolymers, gelatin, being the collagen hydrolysis fragment, has been increasingly used to develop materials with advantages such as proper optical and barrier performance [4,5]. Despite the desirable features of gelatin products, the inherent nature of pure gelatin materials, e.g., relatively low strength and elongation, can limit the applications of associated materials. To meet the application requirements, different techniques such as blending and chemical modification have been practiced to tailor the performance of specific biopolymer materials.

The blending of biopolymers with varied properties is an effective way to acquire composite materials with combined advantages from individual components [6]. The utilization of renewable sources for packaging materials, such as hydrocolloids from biological origins, is one of the main trends of the industry. Investigations have developed blending materials using various biopolymers such as konjac glucomannan (KGM) [7], starch [8], curdlan [9], gelatin [10], chitosan [11], hemicellulosic fractions [12], and galactomannan [13]. Among these biopolymers, KGM is a water-soluble polysaccharide having main chains of D-glucose and D-mannose units linked by *β*-1, 4 glycosidic bonds with branches through *β*-1,6 glycosyl units [14,15]. KGM has excellent film-forming ability [16], proper mechanical features, and high hydrophilicity, making it suitable for the development of KGM/gelatin composites with improved practical performance. 

Earlier findings show that KGM and gelatin have conditional miscibility [10], and transparent KGM/gelatin blend films could be obtained [17]. Changing the ratio of KGM–gelatin could alter the heat-seal strength and moisture uptake of blend films [10,17]. When the KGM content was around 30 wt %, the blend film of KGM and gelatin displayed a good tensile strength of 38 MPa [17]. KGM and gelatin could also be used to develop hard capsules [18]. Investigations have explored the rheological properties and the phase separation during the coupling process for mixed gelatin–KGM gels [19,20]. However, though increasing practices on KGM/gelatin materials have been conducted, there is still limited understanding of how the multiple scale structural features of KGM/gelatin films link to their practical features such as mechanical and hydrophilic properties. This insufficient understanding hinders the rational design of related materials with tailored practical characteristics. 

Hence, this study developed KGM/gelatin blend films under different film-forming (i.e., drying) temperatures (25 and 40 °C). Different analytical methods were used to understand the mechanical and hydrophilic characteristics of the composite films through inspecting the evolutions in their multi-scale structural features, e.g., morphology, nanostructure, crystallites, and molecular features. The addition of KGM improved the mechanical properties and hydrophilicity of KGM/gelatin films. Then, the underlying links between the multi-level microstructures and the practical features were discussed. The present results could benefit the rational production of related composites with related practical performance.

## 2. Materials and Methods

### 2.1. Materials

A konjac glucomannan powder having a molecular weight of 9.67 × 10^5^ Da, high viscosity (30,000 mPa·s at 1% *w*/*v*), and a Type B gelatin (*ρ* = 0.6800 g/mL, no bloom number provided) were purchased from Hubei Konson Konjac Technology Co., Ltd. (Wuhan, China) and Shanghai Aladdin Bio-Chem Technology Co., LTD. (Shanghai, China), respectively. The glycerol and sodium azide used was of analytical grade and supplied by Sinopharm Chemical Reagent Co., Ltd. (Shanghai, China) and Tianjin Hedong District Hongyan Reagent Co., Ltd. (Tianjin, China), respectively.

### 2.2. Preparation of KGM/Gelatin Composite Films

Different weight ratios of KGM–gelatin were used to prepare the composite films through a casting method. KGM–gelatin (1.20 g) with different ratios and 0.24 g glycerol plasticizer in 100 mL of distilled water were placed in a three-neck flask at 90 °C with stirring at 600 rpm for 50 min. Sodium azide (1.0 g, 10% *w*/*w*) used as a chemical preservative e was added to this flask. The obtained mixture was stirred for another 10 min to make the sodium azide evenly distributed in the solution. Then, 100 g of the solutions were transferred onto plastic plates with a radius of 15 cm. The plates were dried in an oven under 40 °C for 27 h or 25 °C for 96 h to allow the formation of KGM/gelatin films. In present work, codes like “K_3_G_5_-25” will be used, where “K” denotes KGM, “G” indicates gelatin, “3” and “5” means 3:5 *wt*/*wt* KGM–gelatin, and “25” shows the drying (film-forming) temperature. The resultant films were peeled off from the plates and conditioned at 25 °C before further use. That is, before the characterization, the films were conditioned at a humidity level of 57% (over saturated sodium bromide solution) for 14 days. The films after conditioning had a water content of about 12.5%, and the weight ratio of gelatin to konjac glucomannan in the films was 10:2 *w*/*w*.

### 2.3. Scanning Electron Microscopy (SEM)

According to an earlier report with modifications [21], the fracture surface characteristics of the films were observed using an SEM system (JSM 6390LV, JEOL, Tokyo, Japan) operated at an acceleration voltage of 15 kV. To obtain film samples with fracture surfaces, the films were fully frozen in liquid nitrogen and then fractured. For the observations, the resultant broken samples with fracture surfaces were coated with a thin gold layer under vacuum. 

### 2.4. Attenuated Total Reflectance Fourier-Transform Infrared Spectroscopy (ATR-FTIR)

The ATR-FTIR spectra of the films were recorded using a Nicolet iS10 (Thermo Fisher Scientific, Waltham, MA, USA) spectrometer, and a Nicolet Smart Orbit ATR accessory was used. For all the spectra, a total scan number of 32 was used in a wavenumber range of 4000–400 cm^−1^ under ambient conditions (26 °C) at a resolution value of 4 cm^−1^. The air spectrum was used as the background and subtracted from the film spectra. 

### 2.5. Small Angle X-Ray Scattering (SAXS)

To evaluate the nanoscale structural features of the films, their SAXS patterns were collected on the BL19U2 SAXS beamline at Shanghai Synchrotron Radiation Facility (Shanghai, China). The KGM/gelatin films were placed on a stage supplied by the Facility, and the 2D scattering data were collected for the film samples with an acquisition time of 10 s. The SAXS patterns were converted from the 2D data, and the patterns at a q range of about 0.0040 to 0.20 Å^−1^ were used as the results. The scattering vector, q, was defined as 4πsinθ/λ, in which 2θ represents the scattering angle and λ indicates the wavelength of X-ray beam [22]. The background scattering was recorded on an empty sample cell, and then the data were background-subtracted and normalized. 

### 2.6. X-Ray Diffraction (XRD)

The film samples were placed on the sample stage of the D8 Advance diffractometer (Bruker, Karlsruhe, Baden-Wuertenberg, Germay) to conduct the XRD measurements, following previous descriptions with modifications [23]. The equipment was operated at 40 kV and 30 mA, with the use of a copper target, a graphite monochromator, and a scintillation counter detector. The X-ray wavelength was 0.1547 nm. The Version 11.0 Evaluation Package (Bruker, Karlsruhe, Baden-Wuertenberg, Germany) was used to process the traces to measure the XRD curves for the film samples. The XRD curve of each film was recorded over a 2θ range of 4° to 50°.

### 2.7. Mechanical Properties

A Texture Analyzer (TA. XT Plus, Stable Microsystems, Surrey, UK) was used to inspect the mechanical properties for the films, based on the ASTM-D-882-91 method. Briefly, each of the films was cut into strips with a size of 5 mm × 50 mm; then, the film was placed between grips to determine the mechanical features involving the tensile strength (*σ*_t_) and the elongation at break ($\varepsilon_{b}$). An initial grip length of 50 mm and a cross-head speed of 0.5 mm/s were used. The Texture Expert software was applied to record the curves regarding the force (N) against the deformation (mm). A micrometer was used to measure the film thickness (μm). According to Equations (1) and (2), the *σ*_t_ (MPa) and $\varepsilon_{b}$ (%) values were calculated

*σ*_t_ = *F*/*T *× *W* (1)

(2) $$\;\varepsilon_{b}\;=\;(L-L_{0})/L_{0}\times \;100\%$$

where *F* (N) indicates the maximum force; *T* (cm) or *W* (cm) mean the thickness or the width of the film; *L*_0_ (cm) reflects the original length of the film; *L* (cm) indicates the length after stretching. 

### 2.8. Contact Angle Analysis

An OCA15EC contact angle goniometer (Dataphysics, Filderstadt, Germany) with a sessile-drop method was used to measure the contact angle of diiodomethane (CH_2_I_2_) on the surface of each agar/KGM film. The film placed on the horizontal movable stage, and the 0.7 μL of CH_2_I_2_ was dropped on the surface of the film. The measurements were taken every 1 s to record the variations of contact angles on the film surface. 

### 2.9. Statistical Analysis

The obtained data were presented as means ± standard deviations. A statistical difference level at *p* < 0.05 was used. The statistical analysis of the data was conducted using Microsoft Excel 2010 (Redmond, WA, USA).

## 3. Results

### 3.1. Microscopic Morphology of Fracture Surface

**Figure 1:**
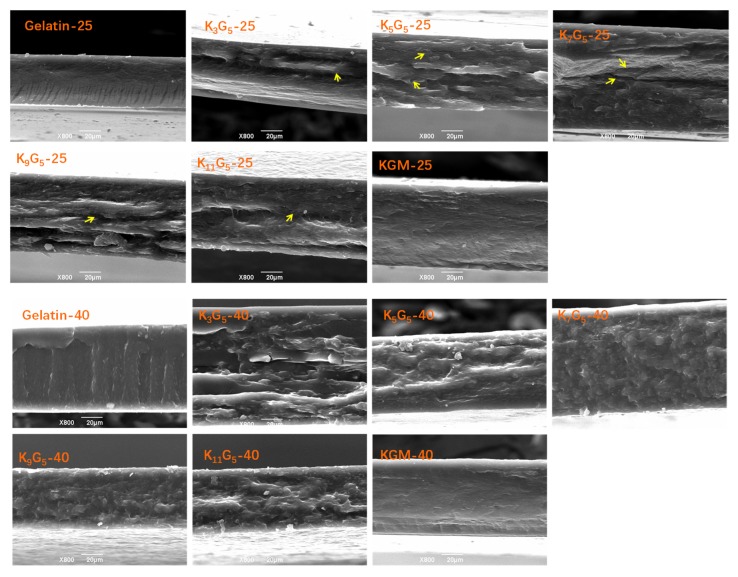
SEM images of konjac glucomannan (KGM), gelatin, and KGM/gelatin films formed under different drying temperatures.

In Figure 1, the SEM graphs present the fracture surfaces of KGM and gelatin films and their composite films prepared under different drying (film-forming) temperatures (25 and 40 °C). For the KGM and gelatin films, a smooth and compact fracture surface could be seen, indicative of a continuous structure matrix of the films (ternary gelatin/konjac glucomannan/glycerol system) on the micron scale. For the KGM/gelatin composite films, the drying conditions at 25 °C led to several notable cracks randomly distributed in the film matrix; a higher drying temperature at 40 °C somewhat eliminated such cracks, giving a relatively compact fracture structure matrix.

### 3.2. ATR-FTIR Spectroscopy

**Figure 2:**
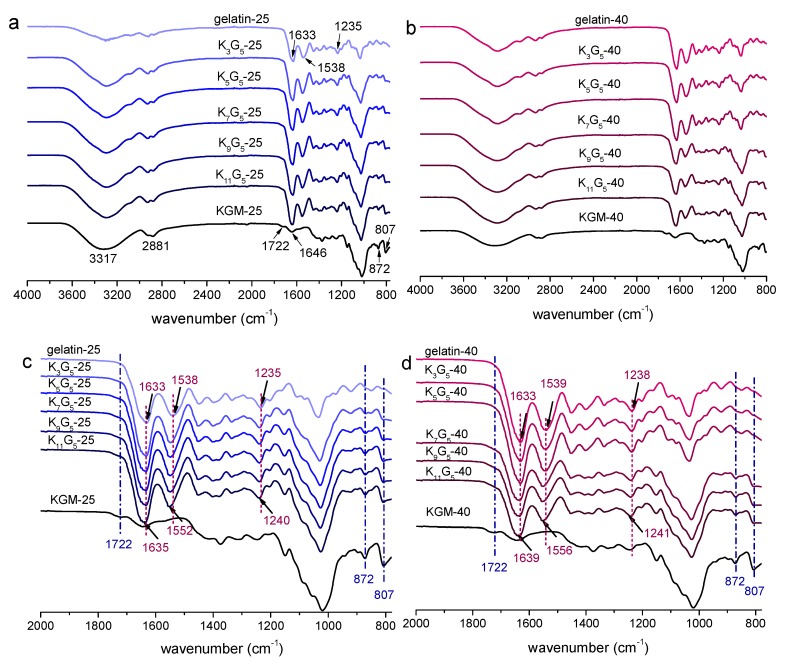
ATR-FTIR spectra of KGM, gelatin, and KGM/gelatin films formed under drying temperature of 25 (full (**a**); enlarged (**c**)) or 40 °C (full (**b**); enlarged (**d**)).

The ATR-FTIR spectra of KGM, gelatin, and KGM/gelatin films are presented in Figure 2. For the KGM film, the peaks at 3317 and 2881 cm^−1^ indicated the stretching of –OH group and methyl C–H, respectively. There were also two bands at 1722 and 1646 cm^−1^ related to the acetyl group and the C–O stretching of the hydroxyl group, respectively, and two peaks at 872 and 807 cm^−1^ associated with the mannose of KGM [7]. The gelatin film showed absorption peaks at 1633 cm^−1^ (amide I, stretching vibration of C=O bond), 1538 cm^−1^ (amide II, coupling of the bending of N–H bond and the stretching of C–N bond), and 1235 cm^−1^ (amide III, vibrations in the plane of C–N bond and N–H bond) [24].

In Figure 2a,b, the KGM/gelatin composite films exhibited the main IR peaks from both KGM and gelatin. As expected, the characteristic peaks of the KGM component at 1722, 872, and 807 cm^−1^ became more appreciable with the increment of KGM content (Figure 2c,d). Irrespective of the preparation temperature used, the absorption peaks of blend films displayed wavenumber positions close to those for the pure KGM and gelatin films, which affirmed negligible evolutions in the chemical structures of component molecules during the film preparation. Note that compared to the gelatin film, the peaks for amide groups (amide I, II, and III) of KGM/gelatin films slightly shifted to higher wavenumbers (Figure 2c,d), and the shifts become more prominent with the KGM content rose. Such a result confirmed the occurrence of interactions between the molecules of the two components [25]. This can be also affirmed by the reduction in ordered structures such as gelatin helix (see XRD results), probably resulting from the fact that the emergence of KGM–gelatin interactions weakened the gelatin–gelatin interactions. 

### 3.3. Nano-Structural Characteristics

**Figure 3:**
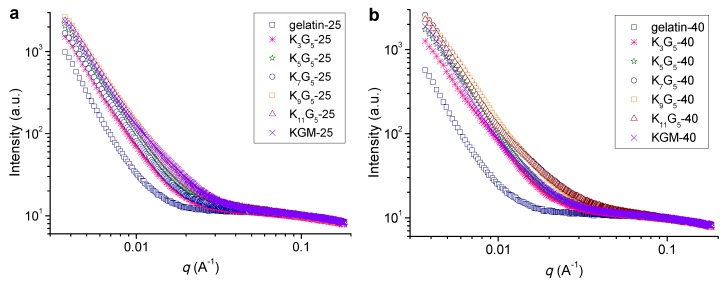
SAXS patterns of KGM, gelatin, and KGM/gelatin films formed at drying temperatures of 25 (**a**) or 40 °C (**b**).

Figure 3 shows the SAXS plots for the films prepared at different film-forming temperatures. At the lower preparation temperature (25 °C), the pure gelatin film did not exhibit detectable features of scattering peak (or broad shoulder peak), indicative of no visible molecular orders on the nanoscales in the film matrix [23]. The addition of KGM into the film led to a sharp increase in the scattering intensity at a q range of below about 0.025 Å^−1^ (K_3_G_5_-25). This phenomenon indicates the formation of nanostructures (length scales above ~25 nm as calculated with the Woolf–Bragg equation [26]) in the blend films, presumably associated with the assembly or aggregates of KGM and gelatin chains. A further increase in the KGM amount tended to more apparently increase the intensity at *q* < 0.025 Å^−1^. While using the higher drying temperature (40 °C), the films also showed no peak or shoulder like scattering manner; and the use of KGM component could cause an increase in the scattering intensity at *q* values up to around 0.030 Å^−1^, revealing the emergence of nanostructures on scales above 21 nm. That is, the increase of film-forming temperature could allow the assembly or aggregation of KGM and gelatin chains into nanostructures at smaller sizes.

### 3.4. Crystalline Structural Characteristics

**Figure 4:**
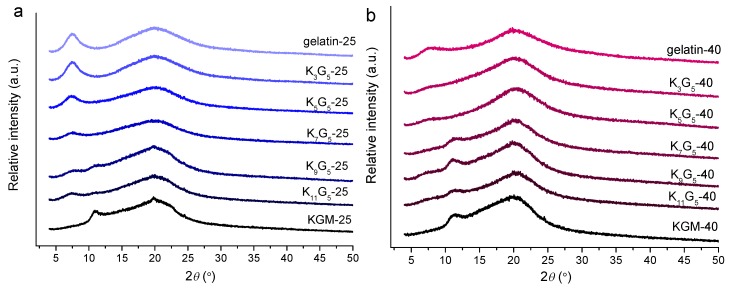
XRD patterns of KGM, gelatin, and KGM/gelatin films formed at drying temperatures of 25 (**a**) or 40 °C (**b**).

The XRD technique is widely used to accurately inspect the crystalline structure of biopolymers [27]. The XRD patterns of the films prepared at different drying temperatures are included in Figure 4. The pure gelatin film showed two peaks at 2θ values of 7.2° and 20.0°, related to the triple-helical crystalline structure [28]. Regarding this, the molecular chains of gelatin are in the sol state in the aqueous film-forming solutions; then, upon cooling and drying, the gelatin chains underwent a disorder-order conformation transition, and assembled to construct the triple-helix structure [29]. The molecular chain assembly into helices and subsequently crystalline components has been reported for various biopolymers such as starch [30,31]. In addition, the pure KGM film displayed two peaks at 2θ of 11.0° and 20.0°, which indicated the existence of KGM ordered structure formed in the course of drying. 

The KGM/gelatin films could exhibit the diffraction peaks of KGM and gelatin. For the composite films formed under the lower temperature (25 °C), the increased KGM amount caused gradual reductions in the gelatin diffraction intensities, and vice versa. This result indicates that the presence of KGM chains could interact with the gelatin molecules, and therefore suppress the assembly of gelatin chains. Consistently, despite that the films at high KGM contents (above 9:5 *wt*/*wt* KGM:gelatin) displayed visible KGM diffractions, the composites with fewer KGM did not show substantial KGM diffractions. Such a phenomenon again confirmed that the presented KGM chains interacted with the gelatin chains, and displayed no apparent signals of KGM crystallites. Furthermore, the increased drying temperature (40 °C) induced reductions in gelatin diffraction intensities and increases in KGM diffraction intensities. That is, the lower temperature drying conditions were favorable for the assembly of gelatin chains, whereas the higher temperature could facilitate the alignment of KGM chains. In addition, all of the film samples did not show notable diffractions from glycerol. These results revealed good compatibility of ternary gelatin/konjac glucomannan/glycerol within the film matrix.

### 3.5. Mechanical Features

**Figure 5:**
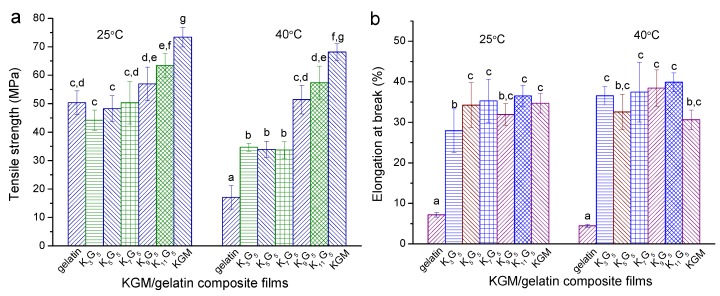
Tensile strength (σt) and elongation at break ($\varepsilon_{b}\;$) for KGM, gelatin, and KGM/gelatin films prepared with drying temperatures of 25 °C (**a**) and 40 °C (**b**). Values with the different lowercase letters letter in each figure differ significantly at *p* < 0.05.

Figure 5 presents the results on the tensile strength (*σ*_t_) and elongation at break ($\varepsilon_{b}$) for the films prepared under different drying temperatures (25 and 40 °C). When the lower drying temperature (25 °C) was used, the increased KGM initially reduced σ_t_ insignificantly and then increased *σ*_t_ apparently, accompanied by generally increased $\varepsilon_{b}$ values. With the higher drying temperature (40 °C), the films could display lower σt values and slightly higher $\varepsilon_{b}$ values as compared to the films with the lower drying temperature of 25 °C; a higher KGM amount in general increased the values of *σ*_t_ and $\varepsilon_{b}$. These evolutions in the mechanical features should be associated with the corresponding changes in the multi-scale structures of the films. 

More specifically, the crystalline components in film matrices can weaken their molecular chain movability, which allows increases in the strength and rigidity and a reduction in the elongation [32]. In contrast, research showed that the elongation for galactomannan/gelatin films was increased, when the mobility of galactomannan chains was enhanced by crosslinking [13]. The polysaccharides having β-1,4 linkages can show higher rigidity than those containing α-1,4 linkages, since the former normally show more difficult conformation transitions with the presence of external forces [33]. With drying temperature at 25 °C and KGM content below 7:5 *wt*/*wt* KGM–gelatin, the reduction in crystallinity level, with the emergence of nanoscale structures (reflected by SAXS), contributed to reducing the film strength but increasing the film extensibility; the small amount of KGM, with KGM–gelatin interactions and without derivatizations (see ATR-FTIR), was not enough to strengthen the films. Consequently, there was a certain reduction in the strength and an increase in the elongation. The further increased KGM chains in the films caused increases in the strength and the elongation, and the certainly-emerged KGM crystallites also tended to strengthen the films. Furthermore, while using the higher preparation temperature (40 °C), the reduced crystalline components of especially gelatin played main roles in weakening the film strength and in increasing the film elongation. The increase of KGM chains, with the increase of KGM crystallites and the presence of nanoscale structures, could be preferential for increasing the strength and the elongation. 

### 3.6. CH_2_I_2_ Contact Angle Features

**Figure 6:**
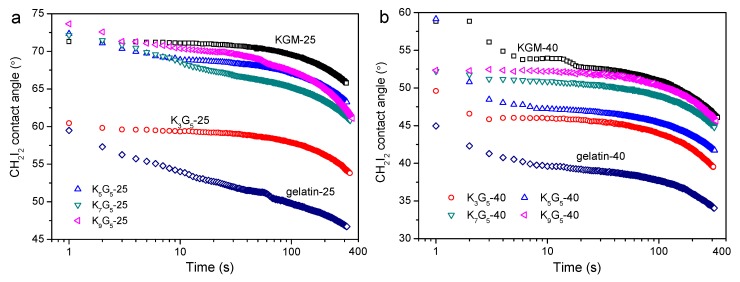
Variations in CH_2_I_2_ contact angle with time for KGM, gelatin, and KGM/gelatin films prepared under drying temperatures of 25 °C (**a**) and 40 °C (**b**).

Since the water contact angle on KGM/gelatin films can change significantly with time rose due to the film hydrophilicity, the contact angle of CH_2_I_2_ was chosen to indicate the hydrophilic or hydrophobic features [34]. Note that the CH_2_I_2_ contact angle may also change slightly after the deposition associated with the CH_2_I_2_ volatilization. Figure 6 presents the CH_2_I_2_ contact angle as a function of time for the films. The results show that the gelatin films and the KGM films, with different drying temperatures, had the lowest and the highest CH_2_I_2_ contact angles, respectively. The increase of KGM content induced a gradual increase in the contact angle values during testing, indicative of increased hydrophilicity mainly resulting from the increase of highly hydrophilic KGM chains, as mentioned previously [18]. The increase in the preparation temperature did not alter the contact angle evolutions as induced by the KGM content. This affirmed that the multi-level structural changes caused by the changed drying temperature did not dominantly affect the changing trend of the film hydrophilicity. Nevertheless, the CH_2_I_2_ contact angles for films with 40 °C drying conditions were lower than those prepared with 25 °C drying temperature, indicating reduced hydrophilicity for the former films. This phenomenon should be related to the enhanced KGM crystallinity with nano-structural aggregations that somewhat weakened the water affinity for predominantly the polysaccharide chains in the films.

## 4. Conclusions

This investigation has inspected the multi-level structural and practical characteristics of KGM/gelatin films with different film-forming temperatures (25 and 40 °C). While using the lower drying temperature (25 °C), the higher KGM:gelation ratio could endow the composite films with reduced gelatin crystallites, accompanied by visible KGM–gelatin molecular interactions, nanoscale structures, and micron-scale cracks, as well as undetected molecular derivatizations. With these structural characteristics, the increased KGM chains led to initially an insignificant reduction and then an increase in the strength, together with a general increase in the elongation. 

The higher film-forming temperature (40 °C) could reduce the strength of the films and only slightly alter the elongation, probably related to the reduced crystallites of especially gelatin. Under this higher temperature, the increased KGM could simultaneously increase the strength and the elongation for the films, presumably due to increased KGM chains and crystallites with the presence of nanoscale structures. In addition, the increase of KGM content tended to make the film more hydrophilic. The changes in the multi-scale structural features of the films did not dominantly affect the changing trend of film hydrophilicity.

The results from this work enable an understanding of the mechanical/hydrophilic features for KGM/gelatin films along with their multi-scale structures, and thus provide fundamental information for the rational design of related composites with tailored practical performance.

## References

[1] Gómez-Guillén M.C., Pérez-Mateos M., Gómez-Estaca J., López-Caballero E., Giménez B., Montero P. (2009). Fish gelatin: A renewable material for developing active biodegradable films. Trends Food Sci. Technol..

[2] Weber C.J., Haugaard V., Festersen R., Bertelsen G. (2002). Production and applications of biobased packaging materials for the food industry. Food Addit. Contam..

[3] Siracusa V., Rocculi P., Romani S., Rosa M.D. (2008). Biodegradable polymers for food packaging: A review. Trends Food Sci. Technol..

[4] Tongnuanchan P., Benjakul S., Prodpran T. (2012). Properties and antioxidant activity of fish skin gelatin film incorporated with citrus essential oils. Food Chem..

[5] Mohammadi R., Mohammadifar M.A., Rouhi M., Kariminejad M., Mortazavian A.M., Sadeghi E., Hasanvand S. (2018). Physico-mechanical and structural properties of eggshell membrane gelatin-chitosan blend edible films. Int. J. Biol. Macromol..

[6] Yu L., Dean K., Li L. (2006). Polymer blends and composites from renewable resources. Prog. Polym. Sci..

[7] Li X., Jiang F., Ni X., Yan W., Fang Y., Corke H., Xiao M. (2015). Preparation and characterization of konjac glucomannan and ethyl cellulose blend films. Food Hydrocoll..

[8] Chen J., Liu C., Chen Y., Chen Y., Chang P.R. (2008). Structural characterization and properties of starch/konjac glucomannan blend films. Carbohydr. Polym..

[9] Wu C., Peng S., Wen C., Wang X., Fan L., Deng R., Pang J. (2012). Structural characterization and properties of konjac glucomannan/curdlan blend films. Carbohydr. Polym..

[10] Li B., Kennedy J.F., Jiang Q.G., Xie B.J. (2006). Quick dissolvable, edible and heatsealable blend films based on konjac glucomannan—Gelatin. Food Res. Int..

[11] Ye X., Kennedy J.F., Li B., Xie B.J. (2006). Condensed state structure and biocompatibility of the konjac glucomannan/chitosan blend films. Carbohydr. Polym..

[12] Cerqueira M.A., Bourbon A.I., Pinheiro A.C., Martins J.T., Souza B.W.S., Teixeira J.A., Vicente A.A. (2011). Galactomannans use in the development of edible films/coatings for food applications. Trends Food Sci. Technol..

[13] Siqueira N.M., Paiva B., Camassola M., Rosenthal-Kim E.Q., Garcia K.C., dos Santos F.P., Soares R.M.D. (2015). Gelatin and galactomannan-based scaffolds: Characterization and potential for tissue engineering applications. Carbohydr. Polym..

[14] Katsuraya K., Okuyama K., Hatanaka K., Oshima R., Sato T., Matsuzaki K. (2003). Constitution of konjac glucomannan: Chemical analysis and 13C NMR spectroscopy. Carbohydr. Polym..

[15] Nishinari K. (2000). Konjac Glucomannan. Dev. Food Sci..

[16] Wu K., Zhu Q., Qian H., Xiao M., Corke H., Nishinari K., Jiang F.T. (2018). Controllable hydrophilicity-hydrophobicity and related properties of konjac glucomannan and ethyl cellulose composite films. Food Hydrocoll..

[17] Xiao C., Lu Y., Gao S., Zhang L. (2001). Characterization of konjac glucomannan–gelatin blend films. J. Appl. Polym. Sci..

[18] Liu Y., Li B., Zhang K., Li J., Hou H. (2019). Novel hard capsule prepared by tilapia (Oreochromis niloticus) scale gelatin and konjac glucomannan: Characterization, and in vitro dissolution. Carbohydr. Polym..

[19] Tomczynska-Mleko M., Brenner T., Nishinari K., Mleko S., Kramek A. (2014). Rheological and thermal behavior of mixed gelatin/konjac glucomannan gels. J. Texture Stud..

[20] Jin W., Xu W., Ge H., Li J., Li B. (2015). Coupling process of phase separation and gelation in konjac glucomannan and gelatin system. Food Hydrocoll..

[21] Qiao D., Tu W., Zhang B., Wang R., Li N., Nishinari K., Riffat S., Jiang F. (2019). Understanding the multi-scale structure and digestion rate of water chestnut starch. Food Hydrocoll..

[22] Zhang B., Zhou W., Qiao D., Zhang P., Zhao S., Zhang L., Xie F. (2019). Changes in Nanoscale Chain Assembly in Sweet Potato Starch Lamellae by Downregulation of Biosynthesis Enzymes. J. Agric. Food. Chem..

[23] Li N., Cai Z., Guo Y., Xu T., Qiao D., Zhang B., Zhao S., Huang Q., Niu M., Jia C. (2019). Hierarchical structure and slowly digestible features of rice starch following microwave cooking with storage. Food Chem..

[24] Ahmad M., Benjakul S., Prodpran T., Agustini T.W. (2012). Physico-mechanical and antimicrobial properties of gelatin film from the skin of unicorn leatherjacket incorporated with essential oils. Food Hydrocoll..

[25] Ma X., Yu J., He K., Wang N. (2007). The Effects of Different Plasticizers on the Properties of Thermoplastic Starch as Solid Polymer Electrolytes. Macromol. Mater. Eng..

[26] Zhang B., Gilbert E.P., Qiao D., Xie F., Wang D.K., Zhao S., Jiang F. (2019). A further study on supramolecular structure changes of waxy maize starch subjected to alkaline treatment by extended-q small-angle neutron scattering. Food Hydrocoll..

[27] Qiao D., Xie F., Zhang B., Zou W., Zhao S., Niu M., Lv R., Cheng Q., Jiang F., Zhu J. (2017). A further understanding of the multi-scale supramolecular structure and digestion rate of waxy starch. Food Hydrocoll..

[28] Peña C., de la Caba K., Eceiza A., Ruseckaite R., Mondragon I. (2010). Enhancing water repellence and mechanical properties of gelatin films by tannin addition. Bioresour. Technol..

[29] Bigi A., Panzavolta S., Rubini K. (2004). Relationship between triple-helix content and mechanical properties of gelatin films. Biomaterials.

[30] Zhang B., Li X., Liu J., Xie F., Chen L. (2013). Supramolecular structure of A- and B-type granules of wheat starch. Food Hydrocoll..

[31] Li N., Niu M., Zhang B., Zhao S., Xiong S., Xie F. (2017). Effects of concurrent ball milling and octenyl succinylation on structure and physicochemical properties of starch. Carbohydr. Polym..

[32] Liu F., Majeed H., Antoniou J., Li Y., Ma Y., Yokoyama W., Ma J., Zhong F. (2016). Tailoring physical properties of transglutaminase-modified gelatin films by varying drying temperature. Food Hydrocoll..

[33] Marszalek P.E., Oberhauser A.F., Pang Y.-P., Fernandez J.M. (1998). Polysaccharide elasticity governed by chair–boat transitions of the glucopyranose ring. Nature.

[34] Qiao D., Li S., Yu L., Zhang B., Simon G., Jiang F. (2018). Effect of alkanol surface grafting on the hydrophobicity of starch-based films. Int. J. Biol. Macromol..

